# SPARC Deficiency Results in Improved Surgical Survival in a Novel Mouse Model of Glaucoma Filtration Surgery

**DOI:** 10.1371/journal.pone.0009415

**Published:** 2010-02-25

**Authors:** Li-Fong Seet, Roseline Su, V. A. Barathi, Wing Sum Lee, Rebekah Poh, Yee Meng Heng, Ed Manser, Eranga N. Vithana, Tin Aung, Matt Weaver, E. Helene Sage, Tina T. Wong

**Affiliations:** 1 Ocular Wound Healing and Therapeutics Group, Singapore Eye Research Institute, Singapore, Singapore; 2 Department of Glaucoma, Singapore National Eye Centre, Singapore, Singapore; 3 Yong Loo Lin School of Medicine, National University of Singapore, Singapore, Singapore; 4 Institute of Medical Biology, Agency for Science, Technology and Research (A*STAR), Singapore, Singapore; 5 Ocular Genetics Group, Singapore Eye Research Institute, Singapore, Singapore; 6 The Benaroya Research Institute at Virginia Mason, Seattle, Washington, United States of America; Tufts University, United States of America

## Abstract

Glaucoma is a disease frequently associated with elevated intraocular pressure that can be alleviated by filtration surgery. However, the post-operative subconjunctival scarring response which blocks filtration efficiency is a major hurdle to the achievement of long-term surgical success. Current application of anti-proliferatives to modulate the scarring response is not ideal as these often give rise to sight-threatening complications. SPARC (secreted protein, acidic and rich in cysteine) is a matricellular protein involved in extracellular matrix (ECM) production and organization. In this study, we investigated post-operative surgical wound survival in an experimental glaucoma filtration model in SPARC-null mice. Loss of SPARC resulted in a marked (87.5%) surgical wound survival rate compared to 0% in wild-type (WT) counterparts. The larger SPARC-null wounds implied that aqueous filtration through the subconjunctival space was more efficient in comparison to WT wounds. The pronounced increase in both surgical survival and filtration efficiency was associated with a less collagenous ECM, smaller collagen fibril diameter, and a loosely-organized subconjunctival matrix in the SPARC-null wounds. In contrast, WT wounds exhibited a densely packed collagenous ECM with no evidence of filtration capacity. Immunolocalization assays confirmed the accumulation of ECM proteins in the WT but not in the SPARC-null wounds. The observations *in vivo* were corroborated by complementary data performed on WT and SPARC-null conjunctival fibroblasts *in vitro*. These findings indicate that depletion of SPARC bestows an inherent change in post-operative ECM remodeling to favor wound maintenance. The evidence presented in this report is strongly supportive for the targeting of SPARC to increase the success of glaucoma filtration surgery.

## Introduction

Glaucoma is the most common cause of irreversible blindness worldwide. The World Health Organisation (WHO) estimates the number of people blinded from glaucoma in 2002 was 4.4 million [1]. The number of people affected worldwide with glaucoma is predicted to be 79.6 million by 2020, of which over 8 million will suffer from bilateral blindness [2]. Elevated intraocular pressure (IOP) is the most important modifiable risk factor for the development and progression of glaucomatous optic neuropathy, which can lead to blindness [3]. Surgery in the form of filtration surgery is considered the most effective method of achieving a consistently desirable low IOP when topical medications are ineffective. This surgery involves the formation of a fistula to maintain blood and aqueous humour flow. A major complication of this surgery, leading to an increased risk of surgical failure and morbidity, is the post-operative wound healing response via obstruction of the fistula by excessive extracellular matrix (ECM) deposition [4]. To limit the post-operative scarring response, physicians often employ anti-proliferative agents to improve surgical outcomes. Mitomycin C (MMC) and 5-fluorouracil (5 FU) are currently used to improve surgical outcomes in glaucoma filtration surgery [5]–[7]. Despite their use intraoperatively as well as postoperatively, a significant failure rate persists and is associated with sight-threatening infections [8], [9]. Therefore, the quest to find an alternative, safe, effective, and targeted anti-fibrotic is ongoing.

SPARC (secreted protein, acidic and rich in cysteine), also known as osteonectin and BM-40, is a 32 kDa calcium-binding matricellular protein that modulates cell-extracellular matrix (ECM) interactions without contributing structurally to the ECM [10], [11]. There is a strong association between elevated expression of SPARC and tissue scarring and fibrosis. Increased expression of SPARC has been observed in fibrotic disorders of the skin [12], [13], lungs [14], kidneys [15], [16], liver [17], and atherosclerotic lesions [18]. Conversely, the SPARC-null (SPARC−/−) mouse exhibited diminished bleomycin-induced pulmonary fibrosis [19] in addition to other characteristics indicative of a compromised maturation and assembly of the ECM. These reports strongly implicate SPARC as a protein involved in tissue remodeling and repair via modulation of ECM organization. However, the targeting of SPARC to reduce not only fibrosis but, more importantly, to preserve wound function that is surgically induced to alleviate a disease condition, has not been described.

In the present study, we show *in vitro* that SPARC−/− conjunctival fibroblasts do not respond to TGF-β2 via increased expression of ECM proteins. Moreover, SPARC−/− cells display lower MMP-2 activity that corresponds to a reduction in the rate of collagen gel contraction. We further demonstrate *in vivo*, by the use of a murine model of glaucoma filtration surgery, that SPARC deficiency can maintain the surgically-induced wound for a longer period of time compared to WT. Specifically, at 14 days post-surgery, 87.5% of the operated SPARC−/− mice retained the blebs compared to 0% of the operated WT mice. We conclude that this observed phenomenon is due to an altered ECM composition in the SPARC−/− wounds which contained less collagen I relative to the WT counterparts. We have therefore provided evidence for the first time of a novel role for SPARC depletion as a promising effective therapeutic method for preservation of wound filtering function in glaucoma filtration surgery.

## Materials and Methods

### Cell Culture

SPARC-null (SPARC−/−) mice and their corresponding WT counterparts (C57Bl6/J) have been described previously [20]. Conjunctival fibroblasts obtained from WT and SPARC−/− mice were cultured as explants in culture medium consisting of Dulbecco's modified Eagle's medium (DMEM) supplemented with 10% fetal bovine serum and Penicillin G-streptomycin sulfate (100 U/ml and 100 µg/ml, respectively) at 37°C in a humidified incubator containing 5% CO_2_. Fibroblasts that migrated out from the tissue were propagated in the same medium. To induce a fibrotic response, we stimulated cells with 2 ng/ml recombinant TGF-β2 (PeproTech Inc., NJ, USA) for 72 h prior to real-time polymerase chain reaction (PCR) assay. All tissue culture reagents were obtained from Invitrogen Corp. (CA, USA) unless otherwise stated.

### RNA Isolation and Expression

Total RNA was recovered with Trizol Reagent (Invitrogen Corp.) according to the manufacturer's recommendations. First-strand cDNA was synthesized with 500 ng total RNA extract and 1 µl of 50 ng/µl random hexamer primer (Invitrogen Corp.) with Superscript III reverse transcriptase (Invitrogen Corp.) according to the manufacturer's instructions. Quantitative real-time PCR (qPCR) was performed in a total volume of 10 µl in 384-well microtiter plates. Each reaction consisted of 1 µl of first-strand reaction product, 0.5 µl each of upstream and downstream primers (10 µM each), 4 µl of Power SYBR Green PCR Master Mix (Applied BioSystems, CA, USA), and 4 ul of DNase-RNase-free distilled water (Sigma-Aldrich Corp., MO, USA). Amplification and analysis of cDNA fragments were carried out by use of the Roche LightCycler 480 System (Roche Diagnostics Corp, Indianapolis, USA). All PCR reactions were performed in triplicate. All mRNA levels were measured as C_T_ threshold levels and were normalized with the corresponding β-actin C_T_ values. Values are expressed as fold increase over the corresponding values for untreated WT control by the 2^−ΔΔCT^ method. The primers used are shown in Table S1.

### Collagen Gel Cultures

Cultured mouse fibroblasts were detached by treatment with trypsin, washed twice with DMEM, and resuspended in FBS at a density of 1.35×10^6^ per ml. Type I collagen (5 mg/ml, Sigma-Aldrich), 10x DMEM, and cell suspension were mixed on ice in a volume ratio of 6∶2.5∶1.5 (final concentration of type I collagen, 3 mg/ml; final cell density, 2×10^5^ per ml). 0.25 ml of the collagen gel suspension was added to each well of a 24-well cell culture dish, and polymerization was induced by incubation at 37°C for 30 minutes. Gels were detached and the wells were topped up with 0.5 ml of culture medium. Quantification of contraction at day-1, -3 and -5 was performed with the Kodak Image Station 4000R (Carestream Molecular Imaging, New Haven, USA). All experiments were performed in triplicate.

### Zymography and Western Blot Analysis

To determine MMP-2 activity, we resolved equal protein concentrations of conditioned medium recovered from contracted collagen gels on day-5 on pre-cast 10% SDS-polyacrylamide slab gels containing 0.1% gelatin (Invitrogen Corp.). After electrophoresis, gels were renatured in renaturing buffer (Invitrogen Corp.) for an hour and then incubated with developing buffer (Invitrogen Corp.) overnight for maximum sensitivity. MMP activity was visualized by staining the gels with Simplyblue Safestain (Invitrogen Corp.) for 1 hour. Images were taken and analysed with the Kodak Image Station 4000R (Carestream Molecular Imaging., New Haven, USA). Densitometric quantitation was performed using the Kodak Image Station 4000R (Carestream Molecular Imaging, New Haven, USA).

For recovery of fibroblasts from the gels, gels were treated as described in a previous report [21]. The dispersed cells were lysed with extraction buffer (20 mM Tris-buffer, pH 7.4, 150 mM NaCl, 1 mM EDTA, 0.5% (volume/volume) Triton X-100, 2 mM MgCl_2,_

1 mM dithiothreitol), and protein content was assessed by the Coomassie Plus Protein Assay Reagent (Thermo Fisher Scientific Inc., IL, USA). Equal amounts of protein were separated by electrophoresis on a 10% SDS gel, transferred to nitrocellulose filters, and probed with a rabbit antibody against MT1-MMP (Abcam plc, Cambridge, UK) followed by a horseradish-peroxidase-conjugated second antibody. Subsequently the blots were developed with SuperSignal West Femto Maximum Sensitivity Substrate (Thermo Fisher Scientific Inc., IL, USA). Blots were stripped and probed with a rabbit antibody against GAPDH (Santa Cruz Biotechnology, Inc., CA, USA). Densitometric quantitation was performed as previously outlined and results were corrected to GAPDH levels, used as a housekeeper, to correct for potential errors in loading.

### Mouse Model of Glaucoma Filtration Surgery

C57Bl/6J WT and SPARC−/− mice were bred and maintained at the Department of Experimental Surgery (Singapore General Hospital, Singapore). All experiments with animals were approved by the Institutional Animal Care and Use Committee (IACUC). The modified filtering surgery in the mouse eye was performed as described in the text. The wound was secured and closed at the limbus by a 10-0 (0.2 metric) Ethilon black monofilament nylon scleral suture (Ethicon, Inc.). Fucithalmic ointment (Leo Pharmaceutical Products, Ballerup, Denmark) was instilled at the end of the procedure. Surgery was performed on eight WT and eight SPARC−/− eyes.

### Detection and Measurement of Blebs

Careful slit lamp and anterior segment-optical coherence tomography (AS-OCT) examinations on subconjunctival blebs were performed daily until post-operative day 3, after which the blebs were observed every 3 to 4 days until postoperative day 14. Slit lamp biomicroscopy was performed with the Nikon FS-3V zoom photo slit lamp (Nikon, Japan). Bleb survival and size of 8 eyes of each genotype were measured by OCT with the Visante™ OCT, Anterior Segment Imaging Model 1000 (Carl Zeiss Meditech Inc., Dublin, CA, USA), and the images were analysed with the Visante™ OCT software. *In vivo* confocal microscopy using the Heidelberg retina tomography HRT3 (Heidelberg Engineering GmbH, Germany) was performed for three eyes of either genotype. *In vivo* confocal images were analysed with the Heidelberg Eye Explorer version 1.5.1 software (Heidelberg Engineering GmbH, Germany).

### Histology and Immunofluorescent Analyses

Control un-operated or operated eyes recovered on day-14 post-surgery were fixed in 4% paraformaldehyde before processing and sectioning with the Microm HM550 (Carl Zeiss Ltd). 5 µm sections were stained with hematoxylin and eosin to visualize tissue morphology. To assess the collagen matrix, we performed picrosirius red staining and visualized the sections by polarization microscopy (Olympus BX51, Olympus, USA) as previously described [22]. For immunofluorescent analysis, we used antibodies specific for SPARC (Santa Cruz Biotechnology, Inc., CA, USA), collagen (Abcam plc, Cambridge, UK), fibronectin (Abcam plc, Cambridge, UK), and MMP-2 (Abbiotec, LLC, CA, USA). Secondary antibodies were conjugated to either AlexaFluro-488 or AlexaFluro-594 (Invitrogen). Sections were visualized under the FV1000 confocal microscope (Olympus, USA). Histological and immunofluorescent analyses were performed on three sets of wounded and unwounded eyes of each genotype.

### Transmission Electron Microscopy (TEM) Analysis

TEM was performed as described previously [23]. For measurement of collagen diameter, at least ten randomly-selected fields of WT or SPARC−/− conjunctiva (magnification, 56,000×) were photographed. The fibril diameters were measured in conjunction with the Image J software.

### Statistical Analysis

Data are expressed as mean ± standard deviation (SD) where appropriate. The significance of differences among groups was determined by the two-tailed Student's t-test with the Microsoft Excel 5.0 software, with significance at a *P*<0.05.

## Results

### SPARC Deficiency Diminishes the Production of ECM Proteins but Not Myofibroblast Differentiation Induced by TGF-β2

**Figure 1:**
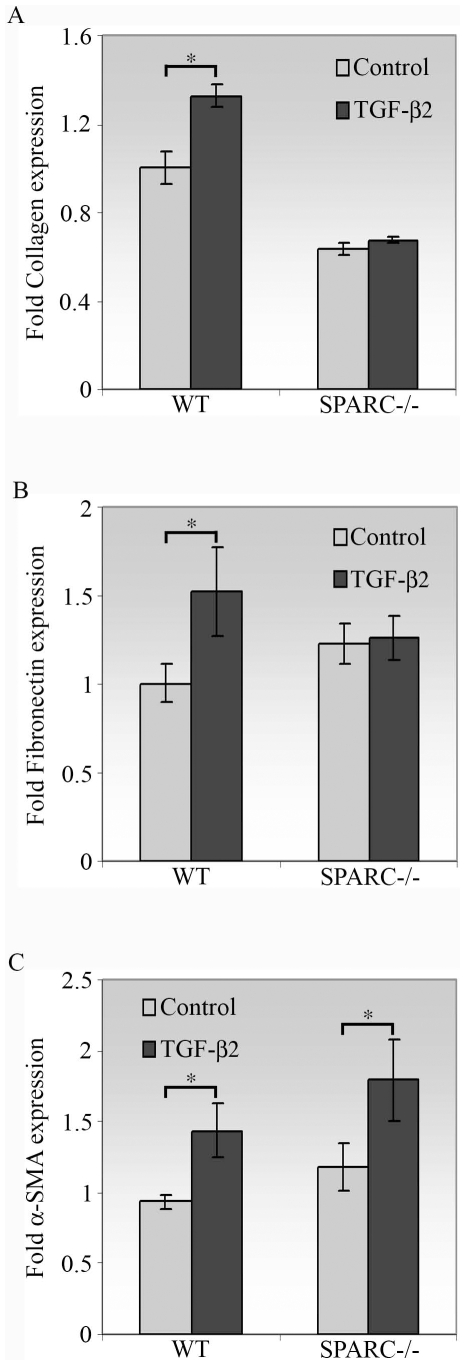
Effect of TGF-β2 on collagen I, fibronectin, and α-SMA mRNA expression. Cells treated with TGF-β2 for 72 h were measured for collagen I, fibronectin or α-SMA mRNA abundance by real-time quantitative PCR. (A) In the presence of TGF-β2, collagen I mRNA was increased in WT (*, *P* = 1.0×10^−5^) but not in SPARC−/− conjunctival fibroblasts. The unstimulated level of collagen I mRNA in SPARC−/− cells was significantly lower than that in WT cells (*P* = 4.3×10^−9^). (B) In the presence of TGF-β2, fibronectin mRNA was enhanced in WT (*, *P* = 0.002) but not in SPARC−/− conjunctival fibroblasts. (C) In the presence of TGF-β2, α-SMA mRNA was increased in both WT and SPARC−/− conjunctival fibroblasts (*, *P*<0.001). The *β-actin* transcript was used for normalization. Data are shown as fold induction compared with unstimulated WT cells. Data shown are representative of three independent experiments.

To assess the effects of SPARC depletion on the conjunctival wound healing response, we performed a series of experiments *in vitro* with conjunctival fibroblasts derived from either WT or SPARC−/− murine eyes. Excess production and deposition of ECM proteins induced by TGF-β are known to play an important role in the development of fibrosis [24]. To evaluate the response of SPARC−/− conjunctival fibroblasts to TGF-β2, we examined the expressions of collagen I and fibronectin. The effect of SPARC depletion on the synthesis of collagen I was striking. First, SPARC−/− fibroblasts appeared intrinsically to produce 1.6-fold lower levels of collagen I in comparison to WT cells (Figure 1A). Second, collagen I was not enhanced by TGF-β2 in the SPARC−/− cells, whereas WT conjunctival fibroblasts exhibited a 1.3-fold spike in collagen I (Figure 1A). Similarly, TGF-β2 induced a markedly enhanced production of fibronectin by WT cells (1.5-fold, Figure 1B). In contrast, SPARC−/− cells showed a negligible increase in fibronectin mRNA when stimulated with TGF-β2 (Figure 1B). Altogether these data imply that SPARC deficiency may alleviate the fibrotic phenotype by reduction of the ECM components collagen I and fibronectin in the presence of TGF-β2. Moreover, the reduced levels of endogenous collagen I indicate that the healing response in the conjunctiva might be altered in SPARC−/− mice.

TGF-β is also an important inducer of epithelial-mesenchymal transition (EMT) *in vivo* and *in vitro* [25]. It is considered to be the major factor for the differentiation of fibroblasts into myofibroblasts, in part due to its augmentation of the expression of the EMT marker α-SMA in fibroblasts. To determine whether depletion of SPARC affects TGF-β2-induced myofibroblast differentiation in conjunctival fibroblasts, we measured the expression of α-SMA in SPARC−/− and WT cells. Induction of α-SMA mRNA was observed in the presence of TGF-β2 in both WT and SPARC−/− fibroblasts (Figure 1C). The similar response observed between SPARC−/− and WT cells indicates that SPARC is not involved in the regulation of TGF-β2-induced myofibroblast differentiation of conjunctival fibroblasts.

### The Absence of SPARC Is Associated with Reduced Fibroblast Contractility Concomitant with Reduced MMP-2 Activity

**Figure 2:**
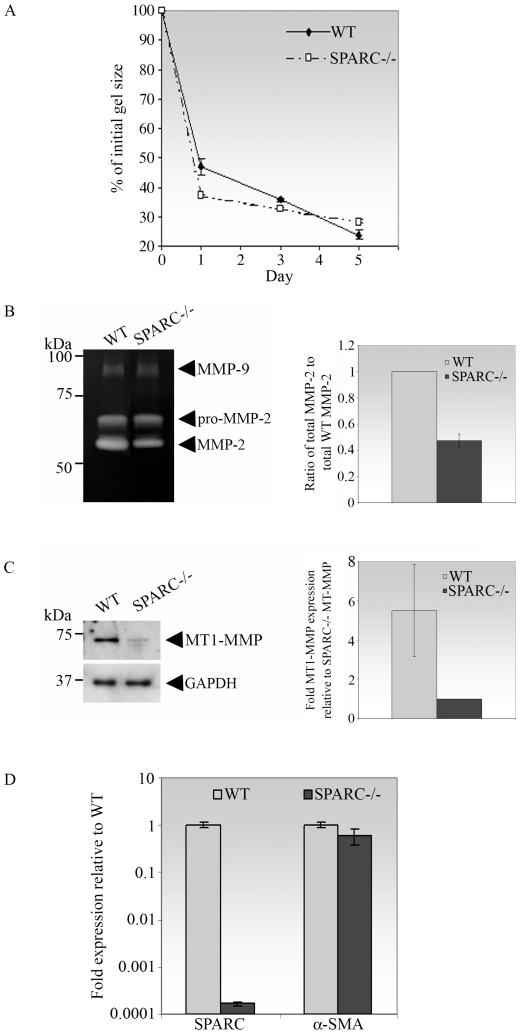
Reduction in the rate of collagen gel contraction by SPARC−/− fibroblasts is associated with a reduction in the expression of MT1-MMP and activity of MMP-2. (A) SPARC−/− conjunctival fibroblasts contracted free-floating collagen gels to a lesser extent, in comparison to that of WT cells. WT or SPARC−/− fibroblasts were seeded in triplicate in collagen solutions which were allowed to polymerize. Gels were immediately detached and gel contraction was digitally photodocumented on day-1, -3 and −5. Gel contraction was measured as a reduction of gel surface area, expressed as a % of the initial gel size measured immediately after detachment. Values are the means of triplicates; bars indicate SD. The data shown are representative of three independent experiments. The rates of contraction from day-3 to day-5 are shown in Table 1. (B) MMP-2 activity was reduced in medium conditioned by SPARC−/− fibroblasts seeded in collagen gels. Left panel, MMP-2 activity in medium conditioned by WT or SPARC−/− fibroblasts seeded in collagen gels for 5 days was analysed by gelatin zymography. Proteolytic activities corresponding to the molecular weights of MMP-9 (92 kDa) and pro- and active MMP-2 (72 and 66 kDa respectively) are indicated by arrowheads. Right panel, densitometric analysis of total MMP-2 (sum of pro- and active MMP-2) showed that the total MMP-2 secreted by SPARC−/− cells was significantly lower that of WT cells (*P* = 2.8×10^−5^). Values shown are the mean of three independent experiments; bars indicate SD. (C) MT1-MMP expression was reduced in SPARC−/− fibroblasts. Left panel, WT or SPARC/− fibroblasts seeded in collagen gels for 5 days were lysed, and 40 µg of each protein preparation was subjected to immunoblotting with antibodies specific for MT1-MMP and GAPDH (control). Right panel, upon normalization with the housekeeping protein GAPDH, densitometry analysis revealed that SPARC−/− fibroblasts produced significantly less MT1-MMP protein compared to WT cells (*P* = 0.04). Values shown are the mean of three independent experiments and a representative Western blot is shown; bars indicate SD. (D) The reduced rate of collagen gel contraction observed in SPARC−/− cells did not involve α-SMA. WT or SPARC/− fibroblasts seeded in collagen gels for 5 days were lysed, and the corresponding SPARC or α-SMA mRNAs were quantified by real-time quantitative PCR. The data confirmed that SPARC expression was sustainably reduced at day-5 in the collagen gels. Values shown are the means of triplicates; bars indicate SD.

Contraction of newly-formed granulation tissue by fibroblasts to bring together the wound edges is an essential process in wound healing [26]. To assess the effect of SPARC deficiency in wound contraction *in vitro*, we utilized the well-established three-dimensional collagen gel contraction model [21], [27]–[29]. In the free-floating gel model, there is no resistance to the contractile force exerted by the cells, and actin stress fibers are minimally involved in the process [30]. In this study, WT or SPARC−/− fibroblasts were cultured in 3D collagen matrices and gel area was measured over 5 days. Contraction, represented by gel area expressed as a percentage of the initial area, was rapid within the first 24 h. Subsequent to the initial 24 h, the rate of contraction decreased and notably, SPARC−/− cells contracted less rapidly than WT cells (Figure 2A). As shown in Table 1, the rate of gel contraction between day 3 and day 5 was 2.7-fold slower for SPARC−/− cells in comparison to WT cells.

**Table 1:** Comparison of the rate of collagen gel contraction from day-3 to -5 between WT and SPARC−/− mouse conjunctival fibroblasts.

	Mean % of initial gel size on day 3	Mean % of initial gel size on day 5	Mean rate of contraction between day 3 and day 5 (% of initial gel size/day)	Fold slower in rate
WT	35.84±0.16	25.53±1.70	−5.16±0.77*	1
SPARC−/−	32.83±0.27	28.99±0.65	−1.92±0.19*	2.69

Values represent the mean % of initial gel sizes ± SD of 3 independent experiments, each performed in triplicate. The rate of contraction between day 3 and day 5 is evaluated as the difference in % of initial gel size between day 5 and day 3 over 2 days. The mean rate of contraction is the average of the rates of contraction of 3 independent experiments. The fold slower in rate is calculated based on mean rate of contraction from day 3 to day 5 of WT over SPARC−/− fibroblasts.

* *P* = 0.01.

Matrix metalloproteinases (MMPs) are required for fibroblast-mediated collagen matrix contraction and ECM remodelling [31]. We therefore examined the activity of MMPs secreted by the mouse fibroblasts seeded in the contracted collagen matrices. Conditioned media were collected from the contracted matrices on day 5 and evaluated by gelatin zymography. Bands of proteolytic activity corresponding to MMP-9 and to pro- and active MMP-2 were detected in all samples (Figure 2B). The level of active MMP-2 in the conditioned media from the SPARC−/− cells was reduced in comparison to that from the WT cells, in stark contrast to the level of MMP-9 activity, which was not obviously different between the two cell types (Figure 2B, left panel). Hence, the inhibitory effect of SPARC depletion on MMP activity appears to be selective for MMP-2. Moreover, measurement of the total MMP-2 indicated that SPARC−/− cells secreted 2.1-fold less of total MMP-2 compared to WT cells (Figure 2B, right panel). Since MMP-2 activation is dependent on the formation of a trimolecular complex consisting of MT1-MMP, pro-MMP-2, and TIMP-2 [32], we proceeded to determine whether the reduction in MMP-2 activity was due to an alteration in the expression of MT1-MMP. Indeed, immunoblots of the cell lysates derived from the contracted matrices after 5 days showed a clear reduction in MT1-MMP in the SPARC−/− relative to the WT cells (Figure 2C, left panel). Densitometric analysis corrected for the housekeeping gene GAPDH confirmed a 5.5-fold reduction in MT1-MMP levels in SPARC−/− fibroblasts (Figure 2C, right panel). We further verified that the free-floating matrices contracted independent of the involvement of actin stress fibers by determining the expression of α-SMA in the cells derived from the 5-day old gels. As shown in Figure 2D, SPARC−/− cells expressed equivalent amounts of α-SMA mRNA as WT cells cultured in the collagen gels. Hence, the slower rate of day 3 to day 5 collagen gel contraction conferred by the SPARC−/− cells is potentially due to a reduction in MT1-MMP and MMP-2 activities.

### SPARC Deficiency Prolongs Bleb Survival in a Murine Model of Glaucoma Filtration Surgery

**Figure 3:**
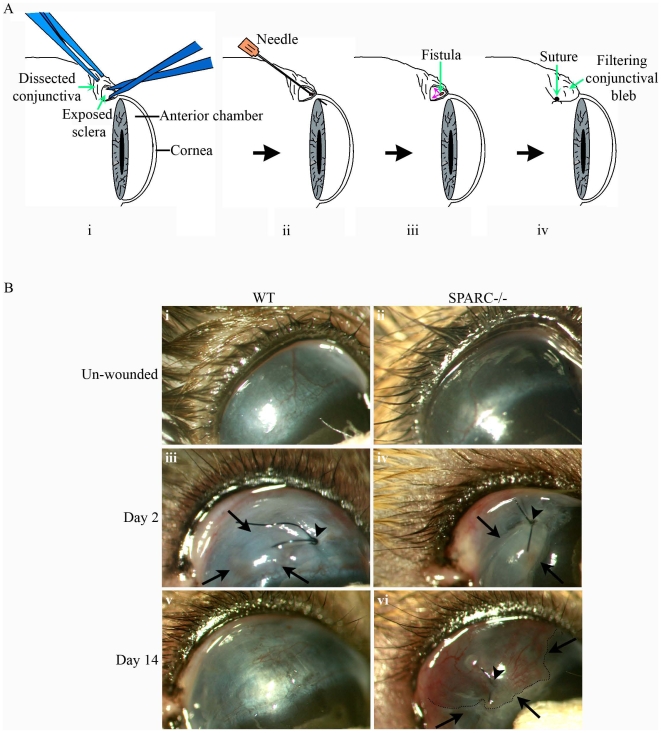
Creation of a filtering bleb in a mouse model of human glaucoma filtration surgery. (A) Schematic diagram of the surgical process involved in the creation of a functioning wound in the mouse conjunctiva. The conjunctiva was first surgically dissected to expose the underlying sclera (i) which was then cannulated with a 30-gauge needle through the sclera into the anterior chamber of the eye (ii) to allow aqueous humor to escape (small red arrows, iii). Finally, the conjunctiva was closed by suturing over the newly-created fistula (iv). The conjunctiva overlying the wound site can be observed as a filtering bleb (iv). (B) Slit lamp examination of wound sites revealed the bleb in SPARC−/− conjunctiva 14 days post-surgery. The conjunctiva in the unwounded eye is flat and smooth (i, ii). On day-2 post-wounding, the wound site close to the suture (arrowhead) assumed the appearance of a bleb (demarcated by arrows), indicative of functionality due to the collection of aqueous fluid in the conjunctiva (iii, iv). On day-14 post-surgery, the WT bleb belonging to the same eye as (iii) has completely flattened down to resemble superficially the unwounded conjunctiva (v), whereas the SPARC−/− bleb belonging to the same eye in (iv) remained visibly elevated (vi, outlined by dotted line and demarcated by arrows).

To investigate the effect of inactivation of the *Sparc* gene on functional wound maintenance, we created a murine model of glaucoma filtration surgery. In brief, the conjunctiva was surgically dissected to expose the underlying sclera, after which an incision was made through the sclera into the anterior chamber of the eye to create a fistula (Figure 3A). This surgically-created channel facilitates the outflow of aqueous humor from the anterior chamber into the subconjunctival space, and thereby effectively reduces the IOP. Filtration of fluid is obvious as an elevated conjunctival bleb, which is readily observed by slit lamp microscopy (Figure 3B). The blebs on day 2 post-surgery were visible as raised and swollen areas of the conjunctiva in WT and SPARC−/− eyes (Figure 3B, iii and iv). However, by day-14 post-surgery, all the WT wounded eyes appeared to have lost the blebs as evidenced by the flattened conjunctiva (Figure 3B, v), whereas the majority of SPARC−/− wounded eyes retained the blebs (Figure 3B, vi).

**Figure 4:**
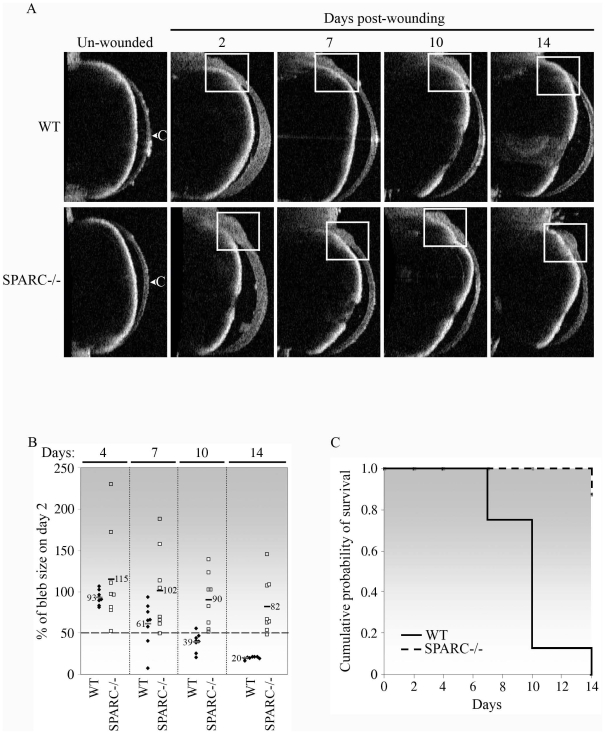
Bleb survival is prolonged in the absence of SPARC. (A) Anterior segment optical coherence tomography imaging of the mouse conjunctiva confirmed bleb survival in the SPARC−/− conjunctiva. The unwounded and wounded eyes on day-2, -7, -10 and −14 were examined. The location of the bleb is indicated by the white box. The bleb was seen to diminish progressively in the WT wounded eye until its virtual disappearance on day-14, whereas the bleb remained visibly present in the SPARC−/− eye. Images shown belong to the same WT or SPARC−/− eye at each of the days examined and are representative of eight eyes of each genotype. C, cornea. (B) Bleb sizes of SPARC−/− mice were larger than those of WT animals. Each bleb at the indicated day post-wounding was measured centrally in at least five OCT imaging fields, and the mean size was expressed as a percentage of the initial size on day-2 post-surgery. Each symbol represents a single eye (♦, WT, n = 8; □, SPARC−/−, n = 8). The horizontal bars and numbers indicate the mean % bleb sizes. (C) Kaplan-Meier curve showing that targeted inactivation of SPARC resulted in increased bleb survival in comparison to that observed in WT eyes. Individual eyes were scored as positive for bleb survival when the bleb size was ≥50% of the initial day-2 bleb as shown in (B). Bleb failure in WT eyes progressed rapidly from 25% (2/8) on day-7 to 87.5% (7/8) and 100% (8/8) on days-10 and -14 post-surgery, respectively, whereas SPARC−/− blebs experienced only 12.5% failure (1/8) on day-14 post-surgery.

Further analysis by anterior segment optical coherence tomography (AS-OCT) was performed to evaluate bleb size and bleb survival. OCT measurements on day-2, -7, -10 and −14 post-surgery confirmed the slit lamp observations (Figure 4A). The bleb in a representative WT wounded eye can be observed to reduce progressively in size until its complete disappearance by day-14 (Figure 4A). In contrast, the bleb in a representative SPARC−/− wounded eye remained visible on each of the indicated days for at least 14 days (Figure 4A). In fact, the measured bleb sizes of the individual SPARC−/− mice (n = 8) were generally larger than those of the WT mice (n = 8) on all the days measurements were taken (Figure 4B). Bleb size is a representation of filtering efficiency of the functioning wound. Hence, the SPARC−/− blebs appeared to be substantially more efficient in the facilitation of aqueous filtration than WT blebs: 37.5% (3/8) on day-4, 50% (4/8) on day-7, 50% (4/8) on day-10 and 37.5% (3/8) on day-14 of the SPARC−/− blebs were larger than their corresponding day-2 blebs, i.e. >100% of the size on day 2. This result is in contrast to 25% (2/8) of the WT blebs on day-4 appearing larger than their corresponding day-2 blebs. Moreover, the largest SPARC−/− bleb size measured was more than 2-fold the size of its day-2 bleb size (230%) in comparison to the largest WT bleb measured, which was only marginally larger in size compared to its size on day-2 (106%) (day 4, Figure 4B).

Although the bleb sizes generally diminished progressively with wound age regardless of the genotype, the decline was notably steeper for the WT eyes. The difference in bleb size between the two genotypes was most prominent on day-14 when the mean bleb size of wounded SPARC−/− eyes was 82% of the corresponding day-2 blebs, compared to 20% in wounded WT eyes (Figure 4B). Bleb sizes, even when expressed as percentages of the corresponding day-2 sizes, can vary significantly between individual eyes. Difficulties in regulation of the wound size during the surgical process, as well as variations in the wound response among individual mice, are likely to contribute to this spread. Judging bleb survival based on bleb size *per se* in this model might therefore not be practical or accurate. Hence, to allow for quantification of bleb survival, we deemed a bleb size that measured ≥50% of the size at day-2 as a functioning bleb, that is, positive for bleb survival whereas a bleb size of <50% was considered a failure and non-survival. Using this criterion, we determined that bleb survival was nil in WT eyes (0/8) and 87.5% in SPARC−/− eyes (7/8) on day-14 post-wounding (Figure 4C). In summary, both slit lamp and OCT measurements indicated that SPARC−/− mice maintained better bleb survival and majority retained at least 50% of the initial bleb size relative to responses observed in WT eyes 14 days post-surgery.

### SPARC−/− Conjunctiva Exhibits a Diffuse Connective Tissue Layer and Responds to Wounding in a Manner Distinct from WT Tissue

**Figure 5:**
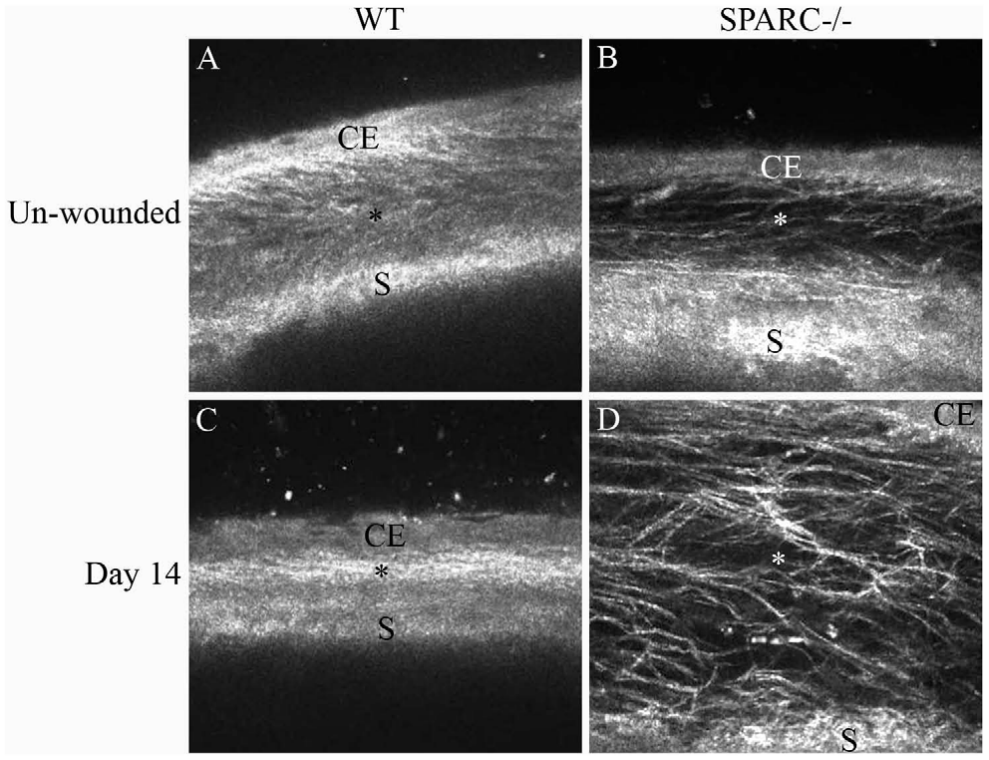
Differential organization of the SPARC−/− versus WT subconjunctival tissue matrix is associated with a distinct wounding response. *In vivo* confocal imaging of the unoperated WT (A) and SPARC−/− (B) conjunctiva revealed an optical difference in the composition and organization of the subconjunctival matrix. On day-14 post-surgery, the WT subconjunctival matrix appeared optically dense and filled with a tightly packed fibrous material (C), whereas the SPARC−/− matrix was expanded and optically diffuse, with a loosely-organized fibrous network (D). Images shown are representative of 3 eyes of each genotype. CE, conjunctival epithelium; *, subconjunctival space; S, sclera.

The mouse conjunctiva is composed of an epithelial covering of two to four layers of keratinocytes resting on a loose collagenous connective tissue that is attached to the underlying sclera. To visualize the conjunctiva *in vivo*, we examined the day-14 eyes by confocal microscopy. As shown in Figure 5A, the normal WT conjunctiva is organized into a distinct outermost epithelial layer followed by a spongy subconjunctival space (stroma) juxtaposed posteriorly to the sclera. The SPARC−/− subconjunctival space appeared less optically dense in comparison to the WT conjunctiva (Figure 5, A and B). At day-14 post-wounding, the WT conjunctival space had collapsed into a compact and optically dense matrix lacking filtering space for aqueous outflow (Figure 5C). In contrast, the wounded SPARC−/− subconjunctival space had expanded at the site of the surgery and was interlaced with optically diffuse matrix material (Figure 5D). The loosely-organized matrix in the latter would be advantageous for the filtration of aqueous fluid through the fistula and percolation through the stromal network.

### SPARC−/− Wounded Conjunctiva Exhibits Deficient Collagen Deposition and Scar Formation

**Figure 6:**
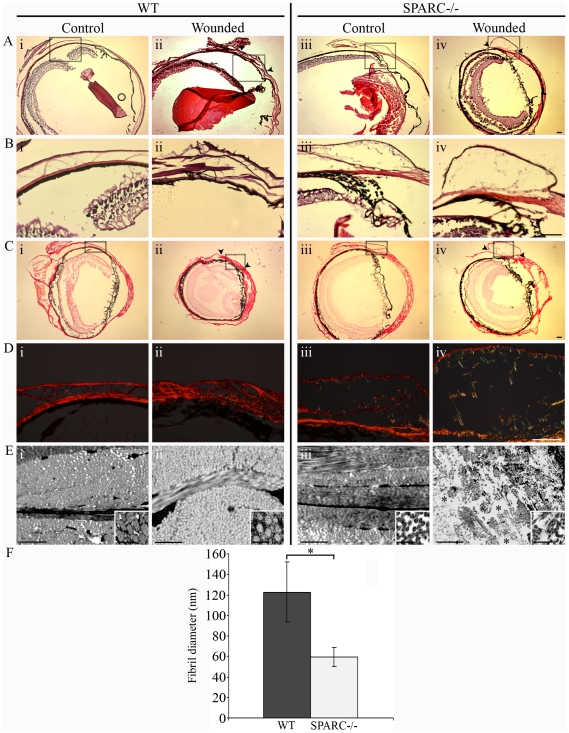
Collagen deposition is altered in the SPARC−/− conjunctiva. (A) Hematoxylin and eosin staining revealed a flattened conjunctiva at the 14 day-old WT wound site (ii, arrowheads), whereas the SPARC−/− wound site was observed as a spongy protuberance in the conjunctiva (iv, arrowheads). (B) Higher magnification of insets (boxed areas in A) illustrate differing thickness in the SPARC−/− (iii and iv) versus WT (i and ii) conjunctival epithelium, independent of wounding. (C) Picrosirius red-stained sections of the same eyes at low magnification and visualized by brightfield microscopy. (D) Higher magnification of insets (boxed areas in C) and polarization microscopy revealed a meshwork organization of collagen fibers in unoperated WT conjunctiva (i) compared to the well-aligned and tightly packed fibers in WT wound (ii), as well as a delicate meshwork in both unoperated SPARC−/− conjunctiva (iii) and operated SPARC−/− bleb (iv). (E) Transmission electron microscopy confirmed a tightly-packed matrix in the WT conjunctival wound (ii), in contrast to the presence of areas of loosely-organized matrix with open spaces in the SPARC−/− wound (*, iv). Ultrastructural analyses also illustrate the intrinsic deposition of smaller collagen fibrils in the unoperated SPARC−/− eye (iii, inset) compared to WT (i, inset). (F) Graphical representation of the mean diameter of collagen fibrils (± SD) from about 10 fields of view of unoperated WT (filled bar, n = 400) and SPARC−/− conjunctiva (open bar, n = 405). *, *P* = 5.1×10^−159^. Bars: (A to D) 100 µm; (E) 1 µm; (E, insets) 200 nm.

We next investigated the physio-anatomical differences underlying the wound response between WT and SPARC−/− eyes. The eyes were cryosectioned on day-14 post-surgery and then subjected to histopathological analyses. The SPARC−/− conjunctival epithelium (Figure 6B, iii) was conspicuously thinner than that in the WT eye (Figure 6B, i). In agreement with data presented in Figure 5, the wounded day-14 WT conjunctiva was characterized by thick strands of fibrous material in the subconjunctival space (Figure 6B, ii). In contrast, the SPARC−/− bleb appeared to be vacuous and punctuated with thin, wispy strands of matrix material (Figure 6B, iv). These observations indicate that collagen deposition might be altered in the SPARC−/− wounds.

To ascertain that collagen deposition in the SPARC−/− versus WT conjunctiva is different, sirus red polarization microscopy was performed. We observed that the thinner conjunctiva in the SPARC−/− mouse was associated with a corresponding thinner collagen layer (Figure 6D, iii), whilst the thicker WT conjunctiva contained densely staining collagen fibers (Figure 6D, i). At the 14 day-old wound site, the WT conjunctiva appeared to be densely compacted with thick, well-aligned collagen fibers resembling a scar (Figure 6D, ii). This arrangement contrasted distinctly with the mechanically efficient basketweave meshwork of collagen in normal conjunctiva (Figure 6D, i). On the other hand, the day-14 bleb in the SPARC−/− eyes contained thin, loosely assembled collagen fibers in an expanded non-collagenous subconjunctival space (Figure 6D, iv). Ultrastructural analysis confirmed that the unoperated WT conjunctiva consisted of tightly-packed collagen fibrils (Figure 6E, i) that accumulated with the absence of conspicuous gaps between fibers after surgical wounding (Figure 6E, ii). In contrast, the unwounded SPARC−/− conjunctiva featured loosely-organized fibrils (Figure 6E, iii) that were interspersed with areas of loose connective tissue in the bleb (Figure 6E, iv). Measurement of the collagen fibrils indicated that the WT conjunctiva contained thicker collagen fibrils (Figure 6E, i, inset), with a mean fibril diameter of 122.72±29.33 nm, in comparison to SPARC−/− conjunctiva, which contained two-fold thinner collagen fibrils (Figure 6E, iii, inset) with a mean diameter of 59.50±9.34 nm (Figure 6F). Thus, it appears that the prolonged bleb survival in the SPARC−/− eyes is related, at least in part, to the deposition of thinner, fewer, and more loosely-organized collagen fibrils. Conversely, the apparently flattened wounds in the WT eyes that consisted of dense, parallel bundles of collagen fibrils is reminiscent of scar formation and is likely to be the cause of fistula obstruction with impediment of aqueous outflow leading to bleb failure.

### Expression of ECM Components Is Generally Suppressed in SPARC−/− Wounds

**Figure 7:**
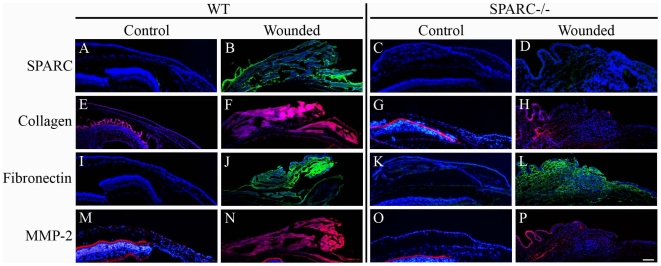
SPARC−/− conjunctival wounds exhibit reduced levels of ECM proteins. Immunofluorescence analyses of normal conjunctiva and wound sites in the WT and SPARC−/− eye revealed that wounding in the WT conjunctiva was associated with increased production of SPARC (B), collagen I (F), fibronectin (J) and MMP-2 (N) in comparison to normal WT conjunctiva (A, E, I, M). The SPARC−/− conjunctiva exhibited no SPARC expression (C, D) and apparently low levels of collagen I (G), and MMP-2 (O) which were not enhanced upon surgical wounding (H, P). There was an apparent increase in fibronectin in the SPARC−/− bleb (L) relative to that in the unoperated SPARC−/− conjunctiva (K). Mouse anti-SPARC and anti-fibronectin antibodies were visualized with anti-mouse secondary antibody conjugated to AlexaFluro-488 (green); rabbit anti-collagen and anti-MMP-2 antibodies were visualized with anti-rabbit secondary antibody conjugated to AlexaFluro-594 (red). Bar, 100 µm.

To identify ECM components in the wounds, we performed immunofluorescence on normal and wounded mouse conjunctiva. In agreement with previous observations that production of SPARC is enhanced at sites of injury, we detected substantial levels of this matricellular protein in the wounded WT conjunctiva (Figure 7B), in contrast to the relatively low level in normal conjunctiva (Figure 7A). The wounded WT conjunctiva exhibited enhanced staining for collagen I (Figure 7F) in comparison to its unoperated counterpart (Figure 7E). Notably, the collagen I immunoreactivity was substantially diminished in the SPARC−/− eyes (Figure 7G) with no apparent increase after wounding (Figure 7H). Staining for fibronectin was also increased after wounding of the WT conjunctiva (Figure 7J), in comparison to the operated conjunctiva in which it was barely detectable (Figure 7I). In the SPARC−/− conjunctiva, wounding resulted in an observable increase in fibronectin deposition (Figure 7L) relative to its unwounded counterpart (Figure 7K). Finally, MMP-2 appeared to be markedly enhanced in WT conjunctiva after wounding (Figure 7N), whereas its levels in the injured SPARC−/− conjunctiva were minimal (Figure 7P).

## Discussion

This study reports a novel therapeutic application of SPARC depletion in the inhibition of conjunctival scarring and the concomitant preservation of bleb survival. Furthermore, we describe here for the first time a murine model of experimental glaucoma filtration surgery. This model illustrates that SPARC plays a pivotal role in ECM organization that is crucial to the maintenance and function of surgically-induced fistula. Beyond its potential application for the evaluation of new therapeutics for glaucoma filtration surgery, this model could be important for the evaluation of post-operative wound healing, with two advantages: (i) the eye is accessible to experimental surgery or application of therapeutics, and (ii), the post-surgical outcome can be easily observed and readily quantified by a variety of available, well-established, and independent imaging techniques, all of which are currently used in the clinical setting such that data generated *in vivo* can be directly compared with those in humans.

Our model has validated previous concepts of wound closure and supports the hypothesis that the primary mechanism of wound closure occurs via connective tissue matrix deposition, that is, by newly-synthesized collagen and other ECM and matricellular proteins [33]. Moreover, the surgical effect of the model in WT eyes reflected the predicted post-surgical outcome of glaucoma filtration surgery in the absence of anti-proliferative agents. Indeed, bleb failure from wound obstruction in glaucoma filtration surgery has been attributed mainly to the accumulation of collagen [34], an effect that is demonstrated in our model.

We have shown in the current study that bleb failure on WT eyes occurred most likely as a result of aqueous outflow obstruction from excessive deposition and organization of ECM in the subconjunctival space. Moreover, the organization of the collagen I fibrils appeared to be mechanically inefficient and likely to contribute further to bleb failure. Hence, our findings support the notion that bleb failure in the WT mice is a result of a massive induction and disorganized deposition of collagenous ECM.

In contrast, the SPARC−/− wound which appeared “open” and filtering, was associated with a reduction in collagen I fibers that were assembled as a loose stromal network in the subconjunctival space. The smaller collagen fibril diameters in the SPARC−/− conjunctiva are also likely to contribute to the “open” wound phenotype. This notion is supported by previous observations in SPARC−/− skin, in which collagen fibril diameters were also reported to be smaller [20], [35], a deficiency that likely contributes to the delayed cutaneous healing observed in another study [36]. Furthermore, altered collagen assembly in SPARC−/− wounds has been correlated with less efficient wound healing in myocardial infarcts, a condition leading to increased incidences of cardiac rupture and failure [37]. Our complementary experiments *in vitro* verified that, in the absence of SPARC, TGF-β2-induced collagen I mRNA was attenuated. Therefore, bleb survival in the SPARC−/− eye can be attributed, at least in part, to diminished collagen I deposition due to a lack of response to TGF-β2 upon wounding.

Several mechanisms have been put forward to explain the reduced expression of collagen I in SPARC−/− cells. One idea is the requirement for SPARC in the post-translational processing of collagen, where it acts as a molecular chaperone to ensure its stability [38]. Indeed, SPARC has been shown to regulate procollagen I processing and collagen fibrillogenesis in skin fibroblasts [35]. This activity could account for collagen stability at the post-translational level and perhaps even for the altered assembly of collagen fibril units in the absence of SPARC. Given our finding that collagen I mRNA levels were perturbed in the SPARC−/− cells, we propose that other mechanism(s) must also be invoked. One possibility is the function of SPARC in the regulation of signaling pathways affecting collagen I expression. Both platelet-derived growth factor and fibroblast growth factor-2 modulate collagen I expression [39], [40], and SPARC is known to affect the activities of these growth factors [10].

The canonical TGF-β signaling pathway involving Smads [41] is known to be involved in the regulation of collagen and fibronectin mRNA transcription [39], [42]. Our observation that SPARC−/− conjunctival fibroblasts failed to increase collagen I expression upon the addition of exogenous TGF-β2 *in vitro* indicates a central involvement of SPARC in the TGF-β signaling pathway. Indeed, expression of TGF-β and SPARC has been shown to be regulated in a reciprocal fashion [43]. The sustained ability of SPARC−/− to respond to TGF-β2 by inducing α-SMA mRNA *in vitro*, points to a role for SPARC in selectively regulating the intracellular canonical TGF-β-induced signaling pathway(s) involved in collagen I and fibronectin expressions in conjunctival fibroblasts.

SPARC is a crucial player in collagen regulation since we found the inhibitory effect of SPARC deficiency on collagen expression was consistently observed in both *in vitro* and *in vivo* experiments. This was not so for fibronectin, where we observed an enhancement of expression in the SPARC−/− wounds *in vivo*, in apparent contradiction to the *in vitro* failure to upregulate its expression in response to TGF-β2. This suggests that the *in vivo* upregulation of fibronectin is a wound response unrelated to TGF-β2 induction. Our observation may also be a reflection of the diverse regulatory pathways involved in regulating fibronectin deposition where SPARC may be dispensible. For instance, fibronectin may be deposited in the wound ECM from the systemic plasma pool [44]. Nevertheless, the evidence suggests that inhibition of collagen I deposition in response to conjunctival injury is sufficient to delay or inhibit wound closure.

That SPARC has been shown to regulate MMP activity [45] supports our data showing that the lack of SPARC is associated with a reduction in MMP-2 activity, concomitant with a decrease in MT1-MMP expression in conjunctival SPARC−/− fibroblasts seeded in the 3D collagen gels. MMPs are known to play critical roles in mediating ECM contraction via its degradation to facilitate the migration of cells and remodel new ECM [32], [46], [47]. Therefore, less MMP activity in SPARC−/− fibroblasts is likely to be a primary cause for the slower rate of collagen gel contraction that we observed in this study. Since contraction enhances wound closure, a slower rate of contraction in the absence of SPARC would in turn delay closure. Moreover, MMP-2 is known to regulate cell migration and angiogenesis, both of which contribute to the development of fibrosis [48]. Hence, reduced MMP-2 expression and activity are likely to help preserve SPARC−/− wound functionality by a delay in wound contraction and inhibition of fibrotic wound healing.

In conclusion, this is the first study demonstrating that SPARC plays a pivotal role in post-operative subconjunctival scarring and that its loss can preserve the presence of a filtering bleb. Inhibition of SPARC expression should be considered as a therapeutic tool to improve glaucoma filtration surgical outcome. Our future work involves employing the gene silencing technology through the application of SPARC-targeting small interfering RNA (siRNA) to reduce SPARC expression in the surgical wound. We believe that this method will be more effective than using SPARC neutralizing antibodies since our data suggests that the mode of action of SPARC is intracellular rather than extracellular, at least where interruption with TGF-β2-mediated induction of ECM production is concerned. SPARC is probably not involved in regulating the availability of secreted TGF-β2 to its receptors as was suggested previously for TGF-β1 [43] since that would result in a curtailment of all TGF-β2 signaling in SPARC−/− cells. The selective inhibition of TGF-β2-induced expression of collagen I and fibronectin but not α-SMA implies that SPARC affects TGF-β2 signaling intracellularly and that diverging pathways are involved in the regulation of collagen I and fibronectin expressions versus α-SMA expression. Hence, future work involves the development of effective SPARC siRNA(s) as well as efficient delivery systems for it so as to improve the survival of the filtering bleb without the need for anti-proliferatives in glaucoma filtration surgery.

## Supporting Information

Table S1 Primers used with real-time PCR.
(0.02 MB DOC)
