# Supplementary material for: SPARC Deficiency Results in Improved Surgical Survival in a Novel Mouse Model of Glaucoma Filtration Surgery
Source: PLoS One. 2010 Feb 25;5(2):e9415. doi: 10.1371/journal.pone.0009415 (PMC2828474; doi:10.1371/journal.pone.0009415)
Supplement: Table S1 — Primers used with real-time PCR. (0.02 MB DOC) [file pone.0009415.s001.doc]

Table S1. Primers used with real-time PCR

| Gene | Forward primer, 5’-3’ | Reverse primer, 5’-3’ |
| --- | --- | --- |
| β-actin | CCAACCGCGAGAAGATGA | CCAGAGGCGTACAGGGATAG |
| collagen I | CAGCCGCTTCACCTACAGC | TTTTGTATTCAATCACTGTCTTGCC |
| Fibronectin | GCTCATCATCTGGCCATTTT | ACCAACCTACGGATGACTCG |
| α-SMA | ccgaccgaatgcagaagga | ACAGAGTATTTGCGCTCCGAA |
